# ICan: An Integrated Co-Alteration Network to Identify Ovarian Cancer-Related Genes

**DOI:** 10.1371/journal.pone.0116095

**Published:** 2015-03-24

**Authors:** Yuanshuai Zhou, Yongjing Liu, Kening Li, Rui Zhang, Fujun Qiu, Ning Zhao, Yan Xu

**Affiliations:** College of Bioinformatics Science and Technology, Harbin Medical University, Harbin 150081, China; Technische Universität Dresden, Medical Faculty, GERMANY

## Abstract

**Background:**

Over the last decade, an increasing number of integrative studies on cancer-related genes have been published. Integrative analyses aim to overcome the limitation of a single data type, and provide a more complete view of carcinogenesis. The vast majority of these studies used sample-matched data of gene expression and copy number to investigate the impact of copy number alteration on gene expression, and to predict and prioritize candidate oncogenes and tumor suppressor genes. However, correlations between genes were neglected in these studies. Our work aimed to evaluate the co-alteration of copy number, methylation and expression, allowing us to identify cancer-related genes and essential functional modules in cancer.

**Results:**

We built the Integrated Co-alteration network (ICan) based on multi-omics data, and analyzed the network to uncover cancer-related genes. After comparison with random networks, we identified 155 ovarian cancer-related genes, including well-known (*TP53*, *BRCA1*, *RB1* and *PTEN*) and also novel cancer-related genes, such as *PDPN* and *EphA2*. We compared the results with a conventional method: CNAmet, and obtained a significantly better area under the curve value (ICan: 0.8179, CNAmet: 0.5183).

**Conclusion:**

In this paper, we describe a framework to find cancer-related genes based on an Integrated Co-alteration network. Our results proved that ICan could precisely identify candidate cancer genes and provide increased mechanistic understanding of carcinogenesis. This work suggested a new research direction for biological network analyses involving multi-omics data.

## Introduction

With the rapid development of high-throughput technologies, databases like The Cancer Genome Atlas project (TCGA)[1] and the Cancer Cell Line Encyclopedia (CCLE)[2] have provided many high-resolution molecular profiles of the same cancer samples, involving gene expression, copy number, methylation and miRNA expression data. These datasets enabled integrative analyses focusing on the identification of cancer related genes. Human tumorigenesis and progression are driven by the aberrant function of genes that regulate aspects of cell proliferation, apoptosis, genome stability, angiogenesis, invasion and metastasis[3]. A major challenge is to identify the cancer-related genes, especially those that play an important role in the initiation and development of cancer. Identifying such genes will contribute to the further development of personalized medicine[4].

Over the last decade, several methodologies have been proposed for the integration of gene expression and copy number data. These methods can be roughly divided into two categories: stepwise integration and joint methodologies[3]. For example, Akavia et al.[5] developed the "genomic footprint" theory, where they extracted driver genes by a method based on a Bayesian network; however, they neglected the correlation between the genes that are simultaneously altered at multiple levels. Bicciato et al.[6] developed a stepwise method called The Significant Overlap of Differentially Expressed and Genomic Imbalanced Regions (SODEGIR) to identify discrete genomic regions with coordinated copy number alterations and changes at transcriptional levels. Salari et al.[7] developed an R package called DRI to identify mRNAs with concordant copy number to expression relationship. There have also been integrative approaches based on canonical correlation analysis that aimed to quantify the association between copy number and expression[8, 9]. On the whole, such methods represents a bioinformatics procedure for the integrative, gene-position based analysis of CN and GE data that allows the identification of discrete chromosomal regions or genes of coordinated copy number alterations and changes in transcriptional levels. In addition to these methods, Louhimo et al.[10] performed an integrative analysis of copy number, DNA methylation and gene expression data, using CNAmet, to identify genes that are coordinately amplified, hypomethylated and upregulated, or coordinately deleted, hypermethylated and downregulated. Although their work integrated multiple data types, we found that they were just focused on the regions or genes with concomitant CN/GE alteration. and don't investigate the direct or indirect relationship between altered genes.

However, cellular functions are rarely determined by a single gene, but rather by many genes combined in the form of networks or clusters. More than one gene is altered in the progression of cancer, they followed distinct patterns of disruption, and cooperated to contribute to tumor phenotype[11]. For instance, a recent study showed that RSF1 regulates genes involved in the evasion of apoptosis (*CFLAR*, *XIAP*, *BCL2* and *BCL2L1*) and regulates an inflammatory gene (*PTGS2*)[12]. Also, studies have observed that the alterations in cancer tend to occur in closely related modules and communities[13]. Therefore, correlations across multiple levels should be taken into consideration seriously. The studies mentioned above did not attach importance to gene-gene correlations. Some other studies have considered these correlations at different levels; however, the tumor activation/suppression mechanisms they revealed were limited to a single level. They did not consider comprehensively the contribution to cancer development by genomic and epigenomic features. They only investigated a driving force of a gene on a single level for cancer progression. For example, coexpression is the most common type of correlation. In 2005, Sean et al.[14] discovered the relation between the high level coexpression of *JAG1* and *NOTCH1* and the poor prognosis of breast cancer. Moreover, the influence of co-mutations between genes was also studied in relation to disease. In 2010, Yunyan et al.[15] examined the functional association between co-mutated genes; their results provided new insights into the complicated coordinating mechanisms of molecular processes. Recently, to increase the accuracy of candidate gene screening, some researchers also included data of mRNA expression and protein interactions. Bashashati et al.[16] developed the DriverNet algorithm, which is based on gene interaction, and identified rare candidate driver mutations that may disrupt transcriptional networks. Despite these efforts, there is still room for improvement. Integrating multi-omics data will help us to develop in silico models that are closer to reality, improving the accuracy of cancer-related gene identification, and providing a more comprehensive understanding of the molecular pathology of cancer.

In this study, we proposed a framework for constructing an Integrated Co-alteration network (ICan). We integrated protein-protein interaction information and the paired data of copy number, DNA methylation and gene expression in 574 ovarian samples. Canonical correlation analysis (CCA) was used to analyze the correlations across genomic, transcriptomic and epigenetic levels, which is the basis of our network. Notably, our approach can not only identify gene pairs that are co-altered at a single level, but also gene pairs with multi-level co-alteration. We found that *CHEK1*, *IGF1R*, *ISG15*, *MSH3* and *PODXL* were co-altered at the copy number, expression and methylation levels at the same time. A co-alteration network of genes can effectively evaluate the strength of an association between genes at multiple levels. The hub genes in this network suggest intracellular interactions and complex functions. We then performed functional analysis and survival analysis to validate candidate cancer-related genes identified by random walking. After multiple testing correlations, we finally obtained 17 gene alterations with prognostic value.

The canonical correlation analysis method is usually used to analyze the degree of correlation between two groups of variables. Unlike the Pearson correlation coefficient, CCA can effectively reveal the linear dependence of two groups of variables so that we could measure genes' correlation using multiple features. We compared the co-alteration network with the single-factor correlation network (co-expression network, co-CNA network, co-methylation network) from the perspective of modules, and found the modules from the integrative method were more compact and more significant (p-value = 2.2e-16). Functional enrichment analysis of genes in the modules showed that they were enriched for certain functions, including cell apoptosis, cell cycle and cancer pathways.

By researching the cancer-related genes and their interrelations, our work will provide a valuable system-level theoretical basis for diagnosis, treatment and drug design in the field of bioinformatics. Our work highlights the importance of systematic integration, and provides clinic researchers with a new insight into the molecular mechanisms of tumorigenesis and progression.

## Materials and Methods

### Data

The Level 3 dataset of gene expression, copy number and DNA methylation for the same set of ovarian cancer samples (Table 1) were obtained from the publicly available TCGA website (https://tcga-data.nci.nih.gov/tcga/). Gistic2.0 was used to analyze the copy number dataset (Level 3) for the identification of recurrent regions of copy number alteration and the copy number of genes. The beta values of DNA methylation are continuous, ranging from 0 (unmethylated) to 1 (completely methylated). The probe IDs were mapped to Gene symbols with the annotation table for Illumina Human-Methylation27 platform, which detected the methylation level of 27,578 CpG loci located within the proximal promoter regions of transcription start sites of 14,495 genes. If there were multiple probes corresponding to the same gene, we adopted the averaged intensity of these probes as the beta value of the gene and removed the probes with no value or corresponding gene. We selected a K-nearest neighbor-based method that imputes missing values in gene expression profiles, which was implemented by an R package (impute). In addition, we have added a list of the samples into supplementary material (see S1 Table).

**Table 1:** Data sets of ovarian carcinoma from TCGA.

Data type	Platform	Samples
gene expression	UNC_AgilentG4502A	574 (8 normal)
copy number	BI_Genome_Wide_SNP_6	574 (8 normal)
DNA methylation	JHU_USC_HumanMethylation27	574 (8 normal)
Clinical data	-	506

To integrate HPRD[17], Reactome[18], MSKCC Cancer Cell Map, and the NCI/Nature Pathway Interaction Database[19], Pathway interaction data and protein-protein interaction data were used to establish the initial network. Pathway data sets for Reactome, the NCI/Nature Pathway Interaction Database, and the MSKCC Cancer Cell Map were downloaded in the Simple Interaction Format (SIF) format from Pathway Commons, protein-protein interaction data was downloaded from HPRD. The Human Background Network (HBN) was the unified set of the four dataset. Simultaneously, redundant edges and self-connected edge were removed (Table 2).

**Table 2:** Four curated datasets for constructing Human Background Network (HBN).

Database	The No. of Nodes	The No. of edges
HPRD	9,617	39,184
Reactome	1,999	15,421
MSKCC Cancer Cell Map	583	1,978
NCI/Nature Pathway Interaction Database	2,233	18,702
ALL	9,195	65,720

We acquired HPRD interactions from the HPRD website (http://www.hprd.org/). Pathway data sets were obtained from Pathway Commons(http://www.pathwaycommons.org/about/)

The HBN we built consists of genes and interactions in the forms of nodes and edges. The interaction reflect the functional associations between two genes, such as a physical interaction, or an indirect interaction via the common pathway.

**Fig 1:**
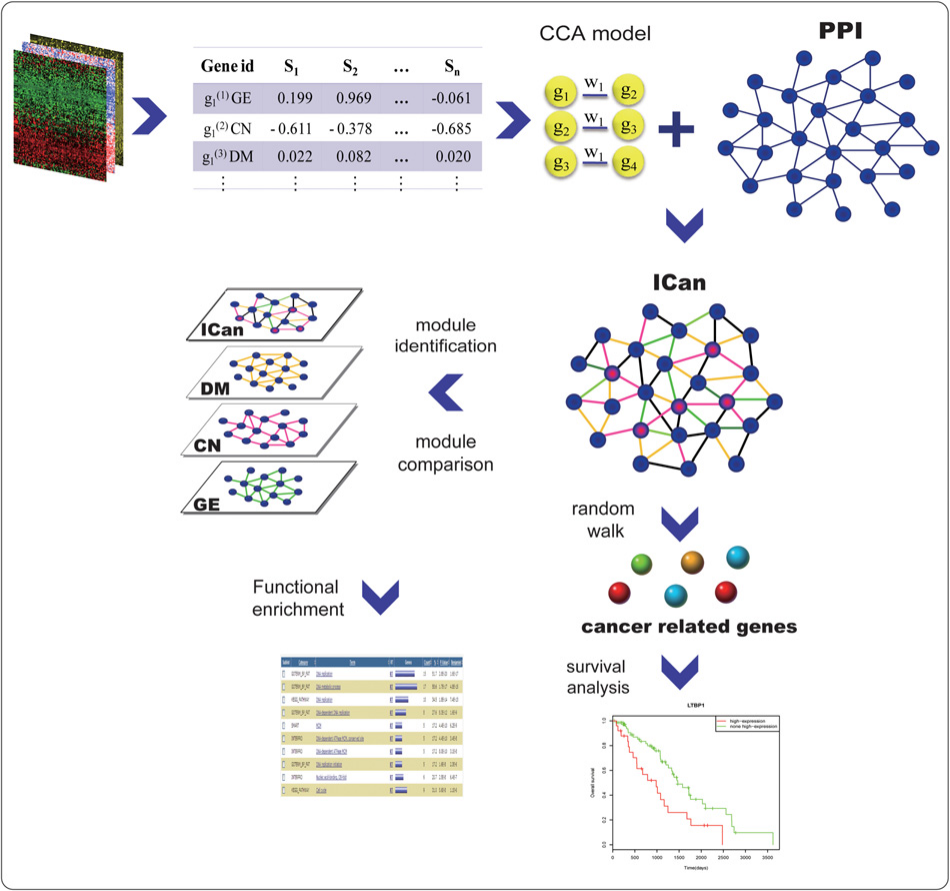
Workflow of the proposed method to identify cancer related genes and functional modules.

We acquired 973 seed genes (S2 Table) from four well-established cancer- and disease-related gene databases: Cosmic[20], GAD[21], OMIM[22] and phenopedia[23]. Ovarian cancer seed genes were defined as known oncogenes or tumor suppressor genes associated with cancer in the well-known databases. The workflow of our approach is depicted in Fig. 1 and further details are provided in the next section.

### Difference analysis of genes in a single level

Gistic2.0[24] was used to analyze the copy number dataset to identify recurrent regions of copy number alteration and the copy number of genes. We identified a number of recurrent focal somatic copy number alteration (SCNA) events, including 55 significant amplifications and 48 deletion peaks. The SAM[25] algorithm was applied to two sets of ovarian samples (tumor/normal) to identify differentially expressed genes: we identified 549 highly expressed genes and 805 low-expressed genes that were differentially expressed in cancer (fold change > = 2 and false discovery rate (FDR) <0.05). For DNA methylation data, we identified highly significant (FDR<0.005) differentially methylated genes in tumor samples compared to normal samples using the Mann-Whitney-Wilcoxon test, including 1445 hypermethylated genes and 1219 hypomethylated genes.

### The construction of the integrated co-alteration network and performance comparison

To simultaneously use multiple features of genes and establish the correlation between genes at the genome, epigenome and transcriptome level, we designed a framework based on CCA, a statistical method used to analyze the degree of correlation between two sets of random variables. CCA can turn the ordinary correlation between two variables into the canonical correlation between two sets of variables. The purpose of CCA is to seek maximization of the correlation between two linear combinations of the variables[26, 27].

In this work, the features of genes were seen as random variables; the possibility of two genes being co-altered on all levels was then measured by the following procedure.

We defined two genes: g_1_, g_2_. Suppose that *G_1_ = [g_1_^(1)^, g_1_^(2) …^,g_1_^(p)^]^T^*, *G_2_ = [g_2_^(1)^, g_2_^(2)…^,g_2_^(p)^]^T^*, and the two vectors consist of *p* types of information of g_1_ and g_2_. In this study, we set *p = 3*.Take *G*_*1*_ for example: *g* ^*(1)*^ denoted the expression values of g1 in samples, *g*_*1*_^*(2)*^ denoted the copy number values of g1 in samples, and *g*_*1*_^*(3)*^ denoted the methylation values of g1 in samples. Similarly, we can define *G*_*2*_.

Let$G=[{}_{G2}^{G1}]$,

Then the covariance matrix is defined as:$\sum =\mathrm{cov}(G,G)=\left(\begin{array}{cc} \sum {}_{11} & \sum {}_{12}\\ \sum {}_{21} & \sum {}_{22} \end{array}\right)$, in which each element is calculated by formula (1).

$$\sum ij=cov(G_{i},G_{j})=E[(G_{i}-\mu_{i})(G_{j}-\mu_{j})]$$ (1)

We use the correlation of linear combination of vectors (namely a^T^G_1_, b^T^G_2_) to measure the linear relationship between G_1_ and G_2_.

The construction of ICan was implemented by seeking the maximum correlation coefficient between *U = a* ^*T*^ *G*_*1*_ and *V = b* ^*T*^ *G*_*2*_

$$\underset{a,b}{max}\;corr(U,V)=\frac{a^{T}\sum_{12}b}{\sqrt{a^{T}\sum_{11}a}\sqrt{b^{T}\sum_{22}b}}$$ (2)

Solutions to the optimization problem (2) satisfied the conditions: *Var(a^T^G_1_) = 1, Var(b^T^G_2_) = 1*.

Our purpose was to seek the most suitable *a* and *b* such that *corr*(U,V) was the largest. The first pair of linear combinations was called the first pair of canonical variables; their largest correlation *ρ*(U_1_,V_1_) was called the first canonical correlation. Next, if there exists *a*_*k*_ and *b*_*k*_ such that the following conditions were satisfied:

1. $a_{k}^{T}G_{1},b_{k}^{T}G_{2}$was uncorrelated with initially K-1 pair canonical variables;
2. $Var(a_{k}^{T}G_{1})=1,Var(b_{k}^{T}G_{2})=1;$
3. The correlation coefficient between $a_{k}^{T}G_{1}$and$b_{k}^{T}G_{2}$ is the largest.

$U_{k}=a_{k}^{T}G_{1},\;V_{k}=b_{k}^{T}G_{2}$were called the first K pair of canonical variables and *ρ(U_k_,V_k_)*was called the first K canonical correlation. In this study, we set K = 3. The Rayleigh quotient matrix:$R=\sum_{11}^{-1/2}\sum_{12}\sum_{22}^{-1}\sum_{21}\sum_{11}^{-1/2}$.

The first correlation coefficient is equal to the square root of the largest eigenvalue *λ*_*1*_ of the matrix R. Similarly, the first K correlation coefficient is equal to the square root of the largest eigenvalue *λ*_*k*_ of the matrix *R*. After that, the linear correlation coefficient (*ρ*_1_,*ρ*_2_,*ρ*_3_) was calculated between every gene pair in the data set.

Canonical correlation is an extension of ordinary correlation; it can measure the correlation between two sets of variables[28]. Compared with using a single data type, it showed more accuracy in the quantification of the linear relationships between genes using their different features[29]. Next, similar to previous works[29], we used the chi-squared test to measure whether the canonical correlation coefficient (*ρ*_1_,*ρ*_2_,*ρ*_3_)[30] was significant.

The null hypothesis is H_0_: *λ*_*k*_ = … = *λ*_*p*_ = 0

Let P_k_ be the *p*-value of the K-th test statistic *T* ^*k*^, with:$T^{k}=-[n-\frac{1}{2}(p+p+3)]\sum_{i=k+1}^{p}log(1-{\hat{\lambda }}_{i}^{2})$, and *T* ^*k*^ ~ $\chi_{\left(p-k)(p-k\right)}^{2}$[29], where *n* is the number of samples. Finally, we used a combination of weights (3) to assign a weight to the edges connecting two genes,

$$\omega =\frac{\sum_{k=1}^{p}\lambda_{k}I(log\;p_{k})}{\sum_{k=1}^{p}I(log\;p_{k})}$$ (3)

Where I(logpk)={0  pk>0.05-logPk  pk≤0.05

The final weight, *ω*, represents the correlation between genes more precisely. *ω* measures the possibility of two genes being co-altered on the level of copy number, DNA methylation and gene expression. We then assigned the weight to the HBN and constructed the integrated co-alteration network referred to as ICan. The method can measure the strength of association between genes on multiple levels. In this work we implemented the CCA method and chi-square-based statistical significance test by the library "CCA" and “Chi-square test” in the R statistical software.

Meanwhile, we computed the Pearson correlation coefficient of the expression profiles (copy number profiles and methylation profiles) between every pair of genes and established a co-expression network(GCE), a co-copy number network(GCC) and a co-methylation network(GCM). This process was also implemented in the R statistical software. To better reflect the performance of our network, we compared ICan and CNAmet, and between three single data networks.

### Identifying candidate ovarian cancer-related genes

Random Walk with Restarts[31] is a sorting algorithm. It simulates the process of walking step by step from seed nodes to direct neighbor nodes; nodes in the network are ranked by the probabilities of reaching the node. Assuming *W* is the adjacency matrix of the ICan and *P*_t_ is a vector whose i-th element holds the probability of arriving at node *i* at step *t*, the random walk was computed by

$$P_{t+1}=(1-r)WP_{t}+rP_{0}$$ (4)

The distribution of values of seed nodes in the initial probability vector *P*_0_ was set as uniform, with the sum of the probabilities equal to 1; *r* represents the probability to restart at seed nodes, which was set to 0.7. After N steps, this probability will reach a steady state, which was determined by the difference between *P*_t_ and *P*_t+1_. We performed the iteration until the L1 norm between them fell below 1E-10. The Random Walk with Restarts probability for all the genes in the network was calculated. We then analyzed the differential alteration of the top 20% genes in the various levels.

### Kaplan-Meier survival analysis for candidate cancer-related genes

A non-parametric Kaplan-Meier estimator was applied to estimate the influence of different factors on survival time. In this work, to explore the possible prognostic value of identified candidate genes, we used the “survival” package in the *R* statistics software. A *p*-value <0.05 and an FDR < 0.25 were used as a cutoffs for statistical significance by the log-rank test.

We investigated the alteration of each gene in the samples, and discretized the three datasets according to the features of oncogenes and tumor suppressor genes, i.e., amplification, overexpression, hypomethylation; and the reverse: deletion, low expression and hypermethylation, respectively. For copy number data, we adopted the results of GISTIC2.0 discrete copy number calls. The samples were classified as gene homozygous deletion (-2) or amplification (1/2). For the gene expression data, we calculated the mean value and standard deviation (SD) for each gene: the values that were higher than mean + SD were considered overexpression. Conversely, the values that were lower than the mean—SD were considered low expression. For the DNA methylation data, we set the threshold based on empirical analysis of the beta value distributions: a beta value less than 0.2 was regarded as hypomethylation; a value more than 0.8 was regarded as hypermethylation.

### Identifying functional modules for ICan

We identified functional modules from ICan and constructed three single-level networks using MCODE[32]. The use of MCODE was preferred for an easier comparison of ICan and the three single-factor networks, as the same modules were identified from the unweighted network. The edge-weighting procedure was performed separately for each network, and the M scores of each module were calculated according to a scoring formula (see Additional file S4 Table for details). A functional enrichment analysis was performed on the candidate cancer-related gene set and the genes inside the module using the DAVID tool[33] (http://david.abcc.ncifcrf.gov/).

## Results

### ICan has the properties of complex networks

The integrated co-alteration network is represented as an undirected weighted graph, where nodes represent genes and edges connecting the nodes represent the correlations of co-alteration between genes. First, making use of human interaction data and pathway knowledge, we established an HBN that comprised 9,195 nodes and 65,720 edges.

In 574 ovarian cancer tumor samples, there are 11,384 genes that are present in all three profiles of copy number, promoter methylation and gene expression. According to CCA, we then calculated the weight between every two genes to measure their linear correlation by the three features. Next, the edges in the network were assigned weights and the genes not contained in molecular profiles were removed. Eventually, we constructed ICan, which comprised 6,345 nodes and 40,125 edges. The closer *ω* is to 1, the higher the correlation between the two genes. In addition, we used the Pearson correlation coefficient for the levels of gene expression, copy number, and DNA methylation to construct three same sized networks.

Network topology plays an important role in the biological functions and information transmission in the network. After analyzing the properties of the network topology, we found that ICan showed a scale-free structure, with a power-law distribution of node degrees. This means that ICan includes only a small number of nodes whose degree is high, suggesting the importance of the hub nodes. We then applied the weighted random walking method to identify hub nodes. This method can effectively optimize candidate disease genes and accurately predict candidate key genes of cancer.

### ICan improves the accuracy of prioritizing candidate cancer-related genes

**Fig 2:**
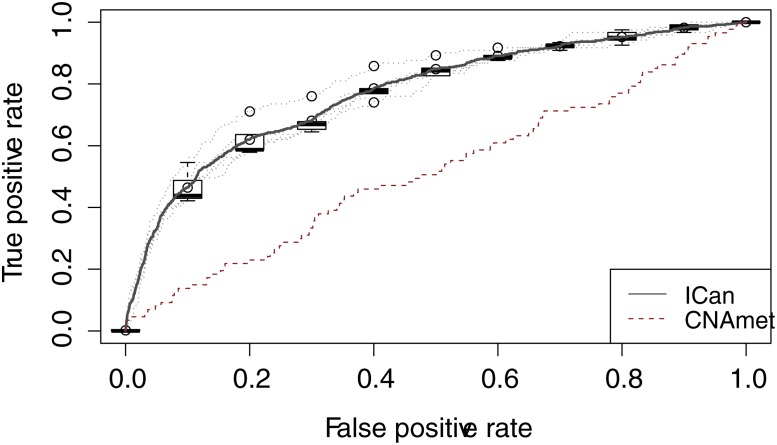
Receiver Operator Characteristic (ROC) Curve for ICan and CNAmet. Black line represents ICan, red dotted line represents CNAmet. Horizontal axis is the false positive rate, the vertical axis is the true positive rate.

ICan contains 604 known ovarian cancer-related genes, which were used as the gold standard to plot receiver operator characteristic curves, and to calculate the area under the curve (AUC). Based on five-fold cross validation, we selected 80% of the genes as seed genes; the remaining 20% were reserved for final validation. To prove the accuracy of our method, using the same data set, we applied the CNAmet method to predict oncogenes and tumor suppressor genes, and compared the outcomes with the ICan outcome. As a result, the AUC value of CNAmet was significantly less than the AUC value of ICan (ICan: the Max AUC = 0.8179; CNAmet: AUC = 0.5183, p-value = 3.158e-14, the first two sheets in S5 Table) (Fig. 2). The significance of the difference of the AUC for two ROC curves was determined by DeLong's test in “pROC package”[34].

**Fig 3:**
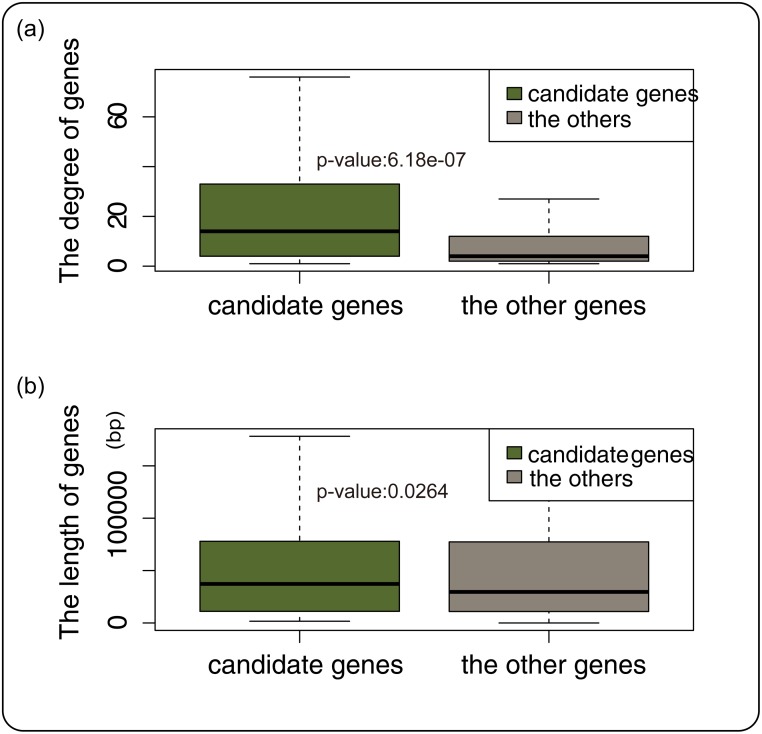
The difference of the node degrees and gene lengths between candidate genes and other genes. In the Fig. 3(a), light green represents candidate genes, gray represents the other genes in ICan, and the vertical axis represents the degree of genes. In the Fig. 3(b), light green represents candidate genes, gray represents the other genes in ICan, and the vertical axis represents the length of genes.

To more accurately predict the cancer-related genes in ovarian cancer, we used a weighted random walking method to calculate the proximity between other nodes and seed genes to determine correlations with oncogenes. This method is often referred to as the “guilt-by-direct-association” principle, by which the genes that are associated with disease genes tend to have similar functions. We randomly chose genes in ICan as seed genes, and compared them with the original results. This process was repeated 1000 times; an adjusted *p*-value below 0.05 was considered significant for cancer-related genes. On the other hand, we compared the difference in the degree[35] and gene length between candidate genes and the other genes. Recent research has shown that a greater gene length often results in more domains in the translated proteins, thus leading to greater interactivity, which means a greater possibility of the gene being cancer gene[36]. The results showed that not only were there significant differences in the gene length of candidate cancer-related genes compared with the other genes (*p*-value = 2.64E-02, Fig. 3, S6 Table), but also the results were similar in terms of gene degree (*p*-value = 6.176E-07).

Finally, we identified 155 candidate cancer-related genes (S7 Table), and analyzed the co-alteration events of these genes in detail. CHEK1, IGF1R and MSH3 were co-altered in common at all three levels; CHEK1, IGF1R, MSH3 and FANCA were co-altered at the copy number and expression levels; and CHEK1, FGF18, IGF1R, IGFBP1, IGFBP2, MSH3, PLAU, RAD51 and EIF2AK2 were co-altered at the level of DNA methylation and expression.

CHEK1, FANCA and RAD51 are involved in the inspection of breakpoints in the cell cycle regulation and repair process, and play important roles either in the p53 signaling pathway or the MAPK signaling pathway. The MAPK signaling pathway is an important cancer pathway; activation of this pathway can promote endothelial cell proliferation and angiogenesis. The newly generated blood vessels could provide more nutrients to tumor cells, accelerating tumor growth and promoting proliferation of cancer cells[37]. MSH3 and IGF1R have important roles in DNA replication, recombination, and repair. Deficiency of mismatch repair, especially loss of expression of the seven main genes (MSH2, MSH3, MSH6, MLH1, MLH3, PMS1 and PMS2), can increase the risk of ovarian cancer[38].

**Fig 4:**
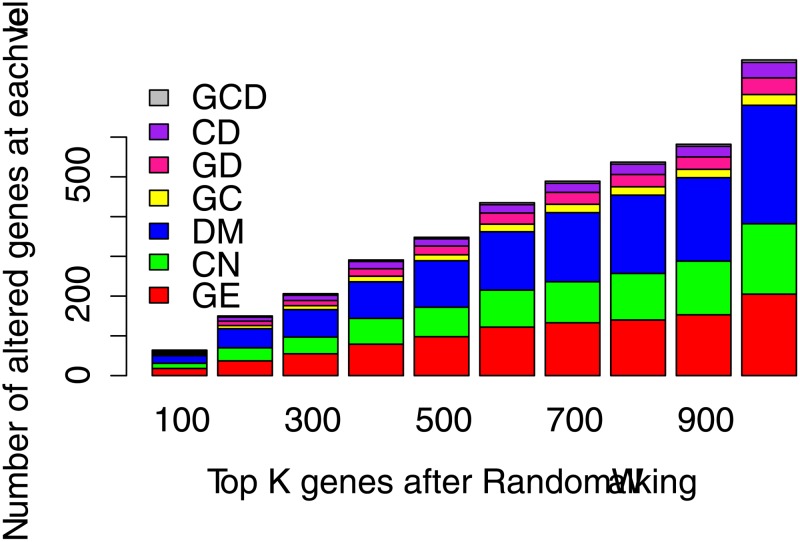
The number of altered genes at each level in TOP100~ALL. We selected TOP 20% gene in ICan by Random Walk, each bar represents the number of differential alteration genes. GE represents the genes that were only were differentially expressed in tumor samples, similarly, CN represents alteration of gene copy number; DM represents DNA methylation; GD represents gene expression and DNA methylation; GC represents gene expression and copy number; CD represents copy number and DNA methylation; GCD represents the genes altered in three features.

In addition, we analyzed the differential proportion of the top 20% genes in ICan by random walking. Fig. 4 shows that the proportion of differential methylation was the highest in each bar among the top 100; however, only two genes have simultaneous differential changes on all three levels. The numbers of genes with only one type of alteration (CNA, differential methylation or differential expression) were 13, 19 and 18, respectively. We found that the number of genes that were differentially altered on multiple levels tended to stabilize after the top 600, which indicated that the probability of these genes is much higher, suggesting a closer relationship with known seed genes.

The alteration of a gene on a single level represented a copy number abnormality, differential expression or differential methylation, respectively (S3 Table, sheet 1–3).

### Novel cancer-related genes of ovarian cancer may affect survival

To estimate the impact of candidate genes on patient survival, and look for genomic and epigenetic genomic features related to patients’ prognosis, we applied survival analysis to estimate the contribution of 6 features for each of the 155 genes (930 total features) on survival time. We identified six significant oncogenic risk factors and 11 significant tumor suppressor factors (S8 Table).

**Fig 5:**
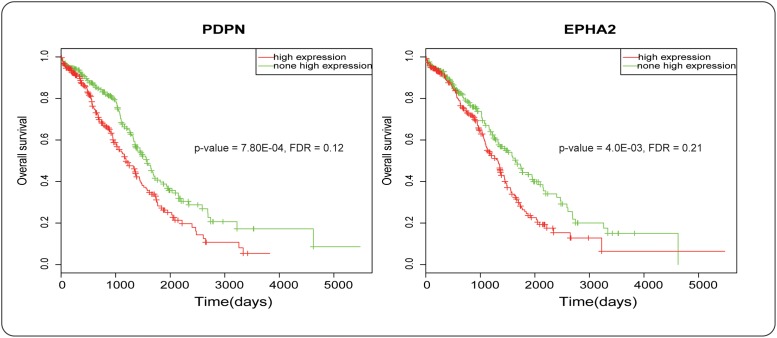
Survival analysis of *PDPN* and *EphA2*. In the left panel, the red line represents the samples with PDPN high-expression and the green line represents the sample slack of PDPN high-expression. In the right panel, the red line represents the samples with EPHA2 high-expression and the green line represents the samples lack of EPHA2 high-expression.

Interestingly, the impact of homozygous deletions of candidate genes on survival was not significant. We speculated that it might result from heterogeneity of the tumor samples. Although the high expression of PDPN did not have a particularly significant impact on poor prognosis (*p*-value = 7.80E-04, FDR = 0.12, Fig. 5). Cancer cells with high PDPN expression have higher malignant potential because of enhanced platelet aggregation, which promotes alteration of cell motility, metastasis and epithelial-mesenchymal transition[39]. Previous studies have shown that overexpression of PDPN in fibroblasts is significantly correlated with a poor prognosis in ovarian carcinoma[40].

We also noted that the overexpression of EphA2 was associated with a shorter patient survival time (Fig. 5). Increased expressions of Eph receptor tyrosine kinases have been implicated in tumor progression in a number of malignancies[41, 42]. It was also observed that abnormal expression of EphA2 could lead to survival of patients with ovarian cancer[43].

### ICan achieved a better modularity

**Fig 6:**
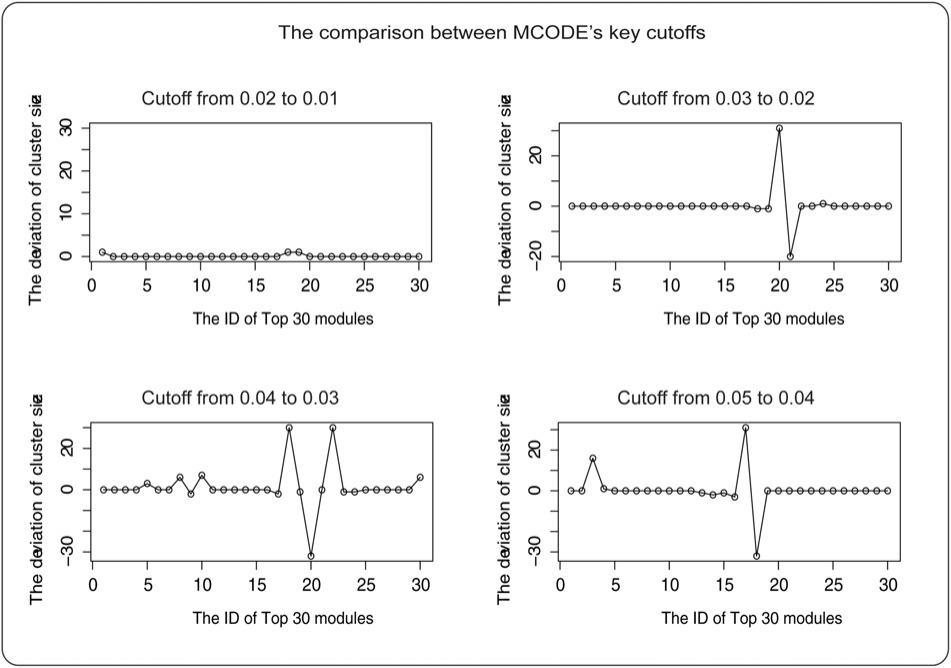
Parameter fitting. The horizontal axis represents the ID of module, the vertical axis represents the deviation of cluster size. It shows the status of cluster sizes as the cutoff changes.

The cutoff is the key parameter of MCODE that influences the size of the module. To select a more appropriate cutoff, we chose 0.01, 0.02, 0.03, 0.04, and 0.05, respectively, for parameter optimization. We found that when the cutoff was 0.02 (Fig. 6), the number of nodes in each module tended to stabilize. Eventually, 133 modules were identified. MCODE does not consider the weight of edges; therefore, we calculated the score *M* for each module using formula (4), and ranked the modules by the scores.

$$M(a)=\frac{\sum_{i=1}^{N-1}\sum_{j=i+1}^{N}\omega_{ij}}{E(a)}$$ (5)

where *a* represents the ID of the module, *E(a)* represents the number of edges in module a, and *N* represents the number of nodes in module *a*.

**Fig 7:**
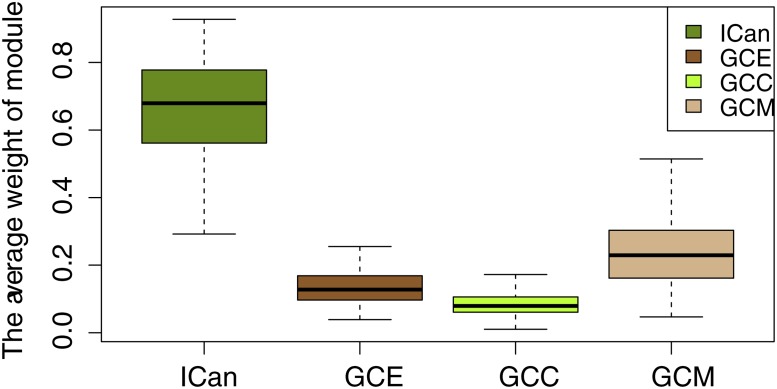
The average weight of modules. The horizontal axis represents the modules of four networks, the vertical axis represents the average weight of modules. ICan represents the integrated co-alteration network; GCE represents gene co-expression network, similarly, GCC represents gene co-CNA network; GCM represents gene co-methylation network.

To further explore the biological functions of ICan, we also compared ICan with the Pearson correlation coefficient method with only a single data type (copy number, methylation or gene expression). The results showed that the weights in the same module were significantly lower than that of the CCA method (*p*-value = 2.2e-16, Fig. 7, S4 Table). The modules of ICan were tighter in structure.

**Fig 8:**
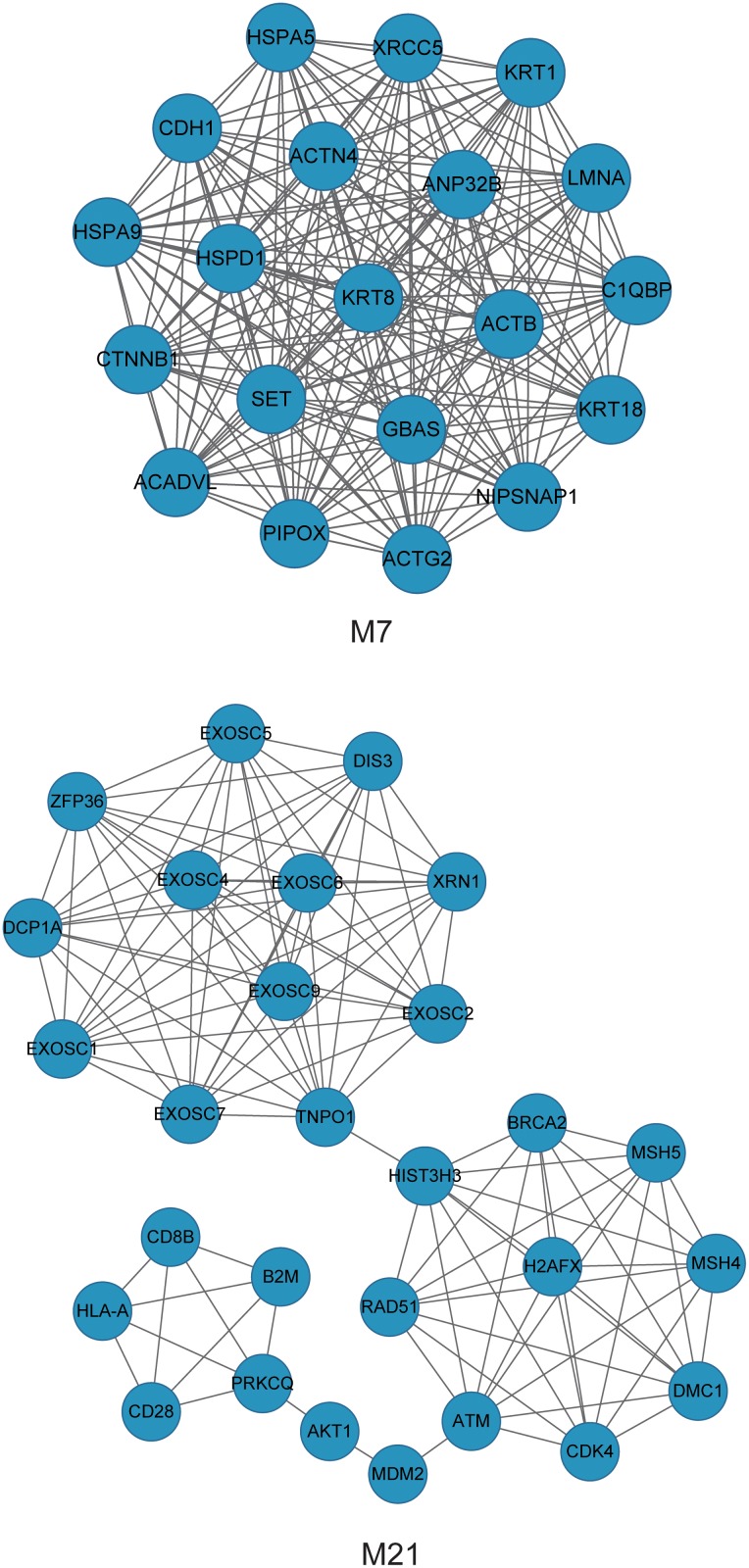
Module 7 and Module 21.

More precisely, the average weight of M7 (Fig. 8) was 0.7210 (CCA), 0.1176 (gene expression), 0.1113 (copy number) and 0.2305 (DNA methylation). We noted that the average weight of ICan was the highest, achieving more than three times the weight of the single-level networks. For instance, *CTNNB1* and *RPA2* were not only co-altered at the level of the copy number, but also at the level of DNA methylation. With respect to a single data type, we uncovered fine correlations between genes. Recent research has also shown that alterations in the genome region of *CTNNB1* and *RPA2* were closely related to the occurrence of ovarian cancer[44, 45]. The M7 module involved 20 genes (*NIPSNAP1*, *ACTN4*, *ACTB*, *PIPOX*, *ACTG2*, *ACADVL*, *KRT8*, *LMNA*, *KRT1*, *KRT18*, *HSPA9*, *HSPD1*, *SET*, *HSPA5*, *XRCC5*, *CTNNB1*, *GBAS*, *C1QBP*, *CDH1* and *ANP32B*) that were significantly enriched to GO terms, including regulation of programmed cell death (2.2E-4), negative regulation of apoptosis (6.5E-4) and adherens junction (6.7E-4) (S9 Table). Among these, *CTNNB1*, *CDH1*, *XRCC5* are important ovarian cancer genes. They are mainly involved in tissue invasion, metastasis and proliferation of cancer.

## Discussion

To compensate for the limitations of former studies, we developed ICan, an integrative method to unearth the cancer related genes. ICan integrates copy number, gene expression and DNA methylation data. This study not only measured differences among genes by a single feature, but also uncovered some critical oncogenes and tumor suppressor genes, such as CCNE1, MYCL1, PIK3R1, FGF2 and other genes (see S4 Table). More importantly, we identified cancer-related genes in human ovarian cancer using the genes' co-alteration characteristics. The co-alteration network built through CCA not only minimized the limitation of a single data type, but also highlighted correlations between simultaneously altered genes, revealing more comprehensive information of disease states. Therefore, ICan improved the accuracy of driver gene prediction.

Compared with previous methods[8, 9], the major improvement in ICan is the integration of a larger panel of driving alterations, including both genetic and epigenetic, such as somatic copy number alteration (SCNA) and methylation. Thus, ICan is valuable for finding closely related patterns between genes in the data sets by three features. By comparison, previous methods studied the correlation between the expression of a single gene and its copy number.

In comparison with the existing method CNAmet, our co-alteration network based on CCA is more effective in the performance of gene-pair scoring. In addition, we also compared ICan with three single networks, In predicting cancer altered genes, the results showed that ICan had a higher accuracy rate (0.8179(ICan), 0.7756(GCM), 0.7779(GCE), 0.7621(GCC)). The network set up linear relationships between genes and the three features, and the weighted random walk algorithm brought in prior cancer gene information. This enabled the evaluation of correlations between nodes and known cancer-related genes. We also compared our network with single-level networks (a co-expression network, a co-CNA network and a co-methylation network). We concluded that multi-layered data integration could systematically enhance our understanding of gene action, and the recognized modules were identified with greater significance. As an example, module M21 (Fig. 8, S4 Table) contained common cancer-related genes such as BRCA2, ATM, MDM2, MSH5, MSH4, RAD51, CDK4, and AKT1, among which BRCA2, ATM and AKT1 directly interact with BRCA1. These genes are all involved in apoptosis.

Our research method depended on prior biological network knowledge, which is used to connect gene pairs. The network integrated the pathways and protein-protein interactions that could link those genes that had no direct interactions but were functionally correlated in the biological network, which is the most important reason for choosing this method. Furthermore, we modified traditional methods of multi-omics data analysis, included the information on gene interactions and capitalized on the correlations between genes to suggest a new research direction of bioinformatics by integrating multi-omics data. However, with the intrinsic limitation of the size of the HBN, some genes participating in the cancer process were filtered out. To make up this efficiency, we could enrich the network information.

Our research is significant for revealing the mechanisms of cancer development and its prognostic impact. We believe that multi-omics data integration will lead to a more systematic understanding of oncobiology. In addition, the variant signatures provide experimental and clinical researchers with an informative resource. Our method can be expanded to other cancers, and new data types may be added in the near future. For example, imbalances of miRNAs also play an important role in cancer development; thus, we could add miRNAs’ regulatory information to the HBN, such that not only could the genes' regulatory information be enriched, but also candidate target genes could be predicted.

## Conclusions

By integrating copy number, methylation, gene expression and protein-protein interaction data of ovarian cancer, we built an integrated co-alteration network (ICan) based on CCA, and identified 155 cancer-related genes, including TP53, BRCA1, RB1 and PTEN; and novel cancer-related genes, such as PDPN and EphA2. Functional annotation and survival analysis suggested the significance of these genes in ovarian cancer. In addition, our method achieved an AUC of 0.8179 in predicting cancer altered genes, which was a better performance than that achieved by CNAmet. The results also indicated that ICan yields better modularity than single-level networks. The genes in the same module participate in proliferation and metastasis of cancer cells.

Our results showed that ICan, built by multi-omics data integration, could aid the precise identification of cancer related genes of ovarian cancer. This study provided a theoretical basis for understanding the mechanism of carcinogenesis and permits searching for new drug targets. The results provided valuable insights into the identification of potential prognostic biomarkers.

## Supporting Information

S1 Table The list of samples. (XLSX)

S2 Table The list of seed genes. The genes in COSMIC and Phenopedia Database were acquired by using “ovarian cancer” as a search keyword, the genes in the other databases (GAD, OMIM) were obtained through mapping the relationships between genes and diseases.
(XLSX)

S3 Table The differential alteration of genes on a single level. Sheet 1: The list of genes with copy-number alteration; sheet 2: The list of differential expression genes; sheet 3: The list of differentially methylated genes.
(XLSX)

S4 Table The scores of four network modules. (XLSX)

S5 Table The data (ICan and individual networks) for plotting the ROC curve and the results of CNAmet. Results of different comparisons can be found on different sheets.
(XLSX)

S6 Table The Degrees and gene lengths of cancer related genes and the others in ICan. (XLSX)

S7 Table The probability values of candidate cancer related genes and random results. (XLSX)

S8 Table The candidate genes by survival analysis with the corresponding p-value by log-rank test. (XLSX)

S9 Table The results of functional enrichment analyses for module M7. (XLSX)
